# Effects of Oral Pumpkin (*Cucurbita pepo* L.) Seed Oil Supplementation on Body Weight, Oxidative Status, Serum IgG Concentrations, and Serum Biochemical Parameters in Weaned Pırlak Lambs

**DOI:** 10.3390/vetsci13090859

**Published:** 2026-08-25

**Authors:** Mehmet Ali Erfidan, Durmuş Fatih Başer

**Affiliations:** 1 Department of Veterinary Medicine, Emirdağ Vocational School, Afyon Kocatepe University, Afyonkarahisar 03600, Turkey; 2 Department of Internal Medicine, Faculty of Veterinary Medicine, Afyon Kocatepe University, Afyonkarahisar 03204, Turkey; dfbaser@aku.edu.tr

**Keywords:** pumpkin seed oil, weaned lambs, functional dietary supplement, immunoglobulin G, lipid metabolism, oxidative stress

## Abstract

Pumpkin (*Cucurbita pepo* L.) seed oil (PSO) is rich in polyunsaturated fatty acids and bioactive compounds, but evidence regarding its biological effects in small ruminants remains limited. This study evaluated the effects of oral PSO supplementation in 45 clinically healthy weaned male Pırlak lambs with a mean initial age of 119.64 ± 35.25 days and a mean initial body weight of 37.55 ± 9.77 kg, allocated to three groups: control (*n* = 9), 0.5 mL/kg PSO (*n* = 18), and 1 mL/kg PSO (*n* = 18). PSO was administered orally once daily for 45 days. Body weight was measured, and blood samples were collected on days 0, 15, 30, and 45. Serum growth hormone, immunoglobulin G (IgG), oxidative status, and selected biochemical parameters were evaluated. PSO supplementation did not significantly affect body weight gain or serum growth hormone concentrations. Total oxidant status was lower in both PSO groups than in the control group on day 30, whereas the oxidative stress index was lower in the 1 mL/kg group than in the control group on day 45. Serum IgG concentrations were higher in the PSO groups throughout the study, although between-group differences were already present at baseline. Serum high-density lipoprotein cholesterol (HDL) concentrations increased over time, particularly in the 1 mL/kg group, whereas low-density lipoprotein cholesterol (LDL) concentrations remained unchanged. Serum glucose concentrations decreased over time and were lower on day 45 than at baseline. No consistent dose-dependent adverse changes were observed in liver or kidney function indicators. Oral PSO supplementation did not improve growth performance but was associated with selective changes in oxidative balance and lipid metabolism without evident hepatic or renal adverse effects. Longer-term studies are required to confirm these effects and clarify their biological significance.

## Simple Summary

Pumpkin seed oil contains natural compounds that may support animal health, but its effects in lambs are not fully understood. In this study, weaned Pırlak lambs were assigned to a control group or to groups receiving pumpkin seed oil daily at 0.5 or 1 mL/kg body weight for 45 days. Supplementation did not improve growth. However, some indicators of oxidative stress were lower, and serum HDL concentrations increased, particularly in lambs receiving the higher dose. IgG concentrations were also higher in the supplemented groups, although these differences were already present at baseline and therefore could not be attributed solely to pumpkin seed oil supplementation. No clear adverse effects on liver or kidney function were observed. These findings suggest that pumpkin seed oil has potential as a dietary supplement for lambs, although further studies are needed to confirm its benefits.

## 1. Introduction

Healthy growth of lambs and their ability to maintain metabolic balance during the weaning period are critically important for flock productivity and economic sustainability in sheep production [1,2]. Weaning is considered one of the most critical transitional stages because incomplete maturation of ruminal function and systemic stress from abrupt dietary changes may compromise homeostatic balance, immune responses, and growth potential in lambs [3]. The metabolic and immune disturbances observed during this period can be monitored using serum metabolite profiles and specific physiological biomarkers [4,5]. Accordingly, the incorporation of natural and bioactive feed additives into diets to optimize immune competence and metabolic health and to support growth performance during weaning and subsequent developmental stages has become an increasingly important area of research [6].

Pumpkin (*Cucurbita pepo* L.) is regarded as a plant resource of high functional value due to its rich phytochemical composition and diverse biological activities [7]. Pumpkin seed oil (PSO) has a characteristic fatty acid profile comprising predominantly linoleic and oleic acids, together with palmitic and stearic acids [8,9]. In addition to this lipid composition, PSO contains bioactive constituents with high antioxidant potential, including tocopherols, lutein, β-carotene, squalene, phenolic compounds, and certain trace elements [10,11,12]. The presence of these constituents has been associated with the antidiabetic, antioxidant, anti-inflammatory, antihypertensive, cardioprotective, and cytoprotective effects of PSO [13,14,15,16,17]. Indeed, studies using different experimental models have reported that PSO or its constituents may reduce oxidative damage, support antioxidant defense systems, and exert beneficial effects on metabolic homeostasis [18,19].

Oxidative stress is an important pathophysiological process resulting from an imbalance between oxidant production and antioxidant defense systems and may lead to cellular damage through lipid peroxidation [20]. Changes in oxidant–antioxidant metabolism during growth and development have been reported to be physiologically important in lambs [21]. In the assessment of this process, integrated parameters such as total antioxidant status (TAS) and total oxidant status (TOS) may provide a more comprehensive assessment of redox status than individual markers such as glutathione peroxidase (GSH-Px), malondialdehyde (MDA), and superoxide dismutase (SOD) [22,23,24]. Moreover, the oxidative stress index (OSI), calculated from TAS and TOS values, is regarded as a more integrated indicator of the net oxidative burden to which the organism is exposed [25,26].

Most studies on PSO have been conducted using monogastric experimental models [27,28,29]. Research on ruminant nutrition has largely been limited to the use of pumpkin seed cake [30,31,32]. In contrast, information on the effects of PSO on growth performance, immunoglobulin G (IgG) concentrations, oxidative balance, and serum biochemical parameters in lambs remains limited [30,33,34]. To address this knowledge gap, the present study aimed to evaluate the effects of orally administered PSO at different doses on body weight gain, IgG concentrations, antioxidant–oxidant balance, and selected biochemical parameters in lambs. The study also aimed to provide scientific evidence on the potential use of PSO as a natural, sustainable dietary supplement for lambs.

## 2. Materials and Methods

### 2.1. Ethical Approval

This study was conducted with the approval of the Afyon Kocatepe University Local Ethics Committee for Animal Experiments (AKUHADYEK), dated 26 March 2025 (Approval No. 49533702-315).

### 2.2. Animals

The study was conducted at the Education, Research, and Application Farm of Afyon Kocatepe University. The study population consisted of Pırlak lambs raised on the farm, which houses approximately 1000 small ruminants. The Pırlak breed is well adapted to the region’s climatic and environmental conditions.

Male Pırlak lambs born between January and April of the same year were included in the study. The lambs were weaned at approximately 100 days of age according to the routine management practices of the institutional farm, and the study commenced after a 15-day post-weaning adaptation period. At the end of the adaptation period, all lambs underwent a comprehensive clinical examination. A total of 50 male lambs determined to be clinically healthy were enrolled in the study. Body weight was used as the primary allocation criterion, while initial age was also considered when allocating lambs to the study groups. The mean initial ages were 114.22 ± 16.65 days in the control group, 122.90 ± 43.00 days in the 0.5 mL/kg PSO group, and 119.11 ± 34.79 days in the 1 mL/kg PSO group. The number of clinically healthy male Pırlak lambs born during the same lambing period and meeting the study eligibility criteria was insufficient to form three equal-sized groups (*n* = 20 each). Therefore, the 50 eligible lambs were initially allocated to the control (*n* = 10), 0.5 mL/kg PSO (*n* = 20), and 1 mL/kg PSO (*n* = 20) groups, with larger supplementation groups to enable more observations for each PSO dose.

During the study, five lambs were excluded because of intercurrent health problems: One lamb in the control group developed caseous lymphadenitis; in the 0.5 mL/kg group, one lamb developed lameness and another developed pneumonia; and in the 1 mL/kg group, two lambs developed lameness. Following these exclusions, the final study population consisted of 45 lambs distributed among three groups: control (*n* = 9), 0.5 mL/kg PSO (*n* = 18), and 1 mL/kg PSO (*n* = 18). No statistically significant difference in initial body weight was detected among the groups, either in the initially enrolled population (*n* = 50) or in the final dataset used for statistical analyses after exclusions (*n* = 45; ANOVA, *p* > 0.05).

### 2.3. Administration Protocol

The study was conducted using a controlled, parallel-group experimental design. The final study population comprised a control group (*n* = 9) and two supplementation groups. To facilitate identification of lambs by group assignment and prevent administration errors, lambs in each group were fitted with differently colored neck collars. Pumpkin (*Cucurbita pepo* L.) seed oil (PSO) was administered individually by oral gavage to the lambs in the two supplementation groups once daily for 45 days at doses of 0.5 and 1 mL/kg body weight, respectively. All administrations were performed at the same time each day. The control group received no vegetable oil. As no previous published study has evaluated oral PSO administration in sheep, the 0.5 and 1 mL/kg doses used in the present study were selected based on the doses employed in a previous study investigating PSO supplementation in rabbits [29].

The daily amount of PSO administered was recalculated using individual body weights measured on days 0, 15, and 30, while individual body weights on day 45 were recorded for the end-of-study evaluation. Throughout the study, the lambs were monitored daily for clinical signs and general health status.

Body weight measurements and blood sampling were performed on days 0, 15, 30, and 45. Serum samples were analyzed for growth hormone (GH), IgG, TAS, TOS, and selected biochemical parameters. The oxidative stress index OSI was calculated from the TAS and TOS values.

### 2.4. Housing and Feeding Conditions

Throughout the study, lambs from all three groups were housed together in a single pen within the same facility and managed under identical husbandry, feeding, and flock management practices to minimize potential confounding effects from environmental factors. All animals had ad libitum access to clean drinking water.

Alfalfa was provided ad libitum as the roughage source. Concentrate feed was offered at 1.1 kg per lamb per day, divided into two equal meals provided in the morning and evening. The concentrate ration was formulated to contain corn (40.5%), barley (30.1%), soybean meal (15.0%), sunflower meal (7.0%), wheat bran (4.0%), limestone (1.5%), dicalcium phosphate (DCP; 1.2%), salt (0.5%), and a vitamin–mineral premix (0.2%). All animals received the same ration throughout the study, and no changes were made to the feeding program.

### 2.5. Sourcing and Analysis of Pumpkin Seed Oil

Pumpkin (*Cucurbita pepo* L.) seeds used in the study were obtained from a commercial producer. The seeds were processed at an authorized facility using cold pressing to obtain pumpkin seed oil. The pumpkin seed oil was stored in a cool, dark place protected from direct sunlight until use.

The fatty acid composition of the resulting oil was analyzed at the Food Control Laboratory Directorate of the Ministry of Agriculture and Forestry. Fatty acid methyl esters (FAMEs) of the pumpkin seed oil were prepared in accordance with the TS EN ISO 12966-2 standard. The fatty acid composition was determined in accordance with the TS EN ISO 12966-4 standard using a gas chromatograph equipped with a flame ionization detector (GC–FID; 7890A GC System, Agilent Technologies, Inc., Santa Clara, CA, USA). The proportion of each fatty acid was calculated as a percentage of the corresponding peak area relative to the total peak area. The fatty acid composition of the pumpkin seed oil is presented in Table 1.

### 2.6. Blood Sample Collection

Blood samples were collected from all lambs on days 0, 15, 30, and 45 of the study in the morning before feeding, while the animals were in a fasted state. Samples were collected aseptically from the jugular vein into 8 mL vacuum tubes containing a gel separator and clot activator.

After allowing the blood samples to clot at room temperature, serum was separated by centrifugation at approximately 2190× *g* for 15 min. The obtained serum samples were transferred into microcentrifuge tubes and stored at −20 °C until analysis. To minimize biological variation associated with circadian rhythm, all blood samples were collected within the same morning time interval for all groups on each sampling day. Serum samples were coded prior to laboratory analyses, and the personnel performing the biochemical and other laboratory measurements were blinded to treatment allocation.

### 2.7. Biochemical Analyses

Serum alanine aminotransferase (ALT), aspartate aminotransferase (AST), alkaline phosphatase (ALP), and gamma-glutamyl transferase (GGT) activities, as well as glucose, blood urea nitrogen (BUN), urea, creatinine, total cholesterol, triglyceride, high-density lipoprotein cholesterol (HDL), low-density lipoprotein cholesterol (LDL), and total protein (TP) concentrations, were measured.

Serum biochemical analyses were performed using a Cobas^®^ 8000 modular analyzer equipped with a c703 module (Roche Diagnostics GmbH, Mannheim, Germany; Hitachi High-Technologies Corporation, Tokyo, Japan). Commercial reagents, calibration procedures, and internal quality-control protocols were used in accordance with the manufacturers’ recommendations. All samples were analyzed in the same laboratory using the same analytical system and under identical analytical conditions.

### 2.8. Oxidant–Antioxidant and ELISA Analyses

Serum TAS and TOS levels were determined using commercial colorimetric assay kits in accordance with the manufacturer’s instructions (Elabscience Biotechnology Co., Ltd., Wuhan, China). Serum IgG and GH concentrations were measured using species-specific commercial ELISA kits: the FineTest^®^ Sheep IgG ELISA Kit and the FineTest^®^ Sheep GH ELISA Kit, respectively (Wuhan Fine Biotech Co., Ltd., Wuhan, China).

Absorbance measurements for the TAS, TOS, IgG, and GH assays were performed using a Thermo Scientific Multiskan^TM^ FC Microplate Photometer (Thermo Fisher Scientific, Vantaa, Finland). Serum samples for the ELISA and TAS/TOS assays were analyzed in duplicate, and the mean of the two measurements was used for statistical analysis. All assays were conducted in accordance with the manufacturers’ protocols, and sample concentrations were calculated using standard curves. Results were expressed as mmol Trolox equivalents/L for TAS, µmol H_2_O_2_ equivalents/L for TOS, mg/mL for IgG, and pg/mL for GH.

### 2.9. Calculation of the Oxidative Stress Index

The oxidative stress index (OSI) was calculated as the ratio of TOS to TAS. Before calculation, TAS values were converted from mmol Trolox equivalents/L to µmol Trolox equivalents/L. OSI was then calculated using the following formula [35,36]:

OSI (arbitrary units) = [TOS (µmol H_2_O_2_ equivalents/L)/TAS (µmol Trolox equivalents/L)] × 100.

### 2.10. Statistical Analysis

All statistical analyses were performed using Minitab 18. Data obtained from the ELISA and biochemical analyses were evaluated according to supplementation group and measurement day. The supplementation factor consisted of three levels: control, 0.5 mL/kg, and 1 mL/kg, whereas the measurement-day factor consisted of four levels: days 0, 15, 30, and 45. Differences among supplementation groups were evaluated separately on each measurement day using one-way analysis of variance. In these analyses, the supplementation group was treated as a fixed factor, and Tukey’s multiple comparisons test was used to identify differences among group means. In addition, a linear mixed-effects model for repeated measures was used to evaluate the effects of supplementation group, measurement day, and the supplementation group × measurement day interaction. Supplementation group, measurement day, and their interaction were included as fixed effects, whereas the individual lamb was included as a random intercept to account for correlations among repeated measurements within the same animal. Variance components were estimated using restricted maximum likelihood (REML), and the Kenward–Roger approximation was used to calculate the denominator degrees of freedom for tests of fixed effects. The *p*-values for the supplementation group × day interaction were presented in the tables as pD × G (pDay × Dose Group). Results were expressed as mean ± standard deviation. Within the same row, means denoted by different uppercase letters indicate significant differences among measurement days within the same supplementation group, whereas within the same column, means denoted by different lowercase letters indicate significant differences among supplementation groups on the same measurement day. Statistical significance was set at *p* < 0.05.

### 2.11. Use of Generative AI

ChatGPT (OpenAI, GPT-5.5, San Francisco, CA, USA) was used solely for grammar and language editing during the preparation of this manuscript. All revisions were reviewed and approved by the authors.

## 3. Results

### 3.1. Study Population and Changes in Body Weight

The study was completed with a total of 45 male lambs. No statistically significant differences in initial body weight were detected among the groups (*p* > 0.05). Although body weight increased progressively in all groups throughout the study, no statistically significant between-group differences were detected at any measurement time point (Table 2).

### 3.2. Serum Oxidative Stress Parameters

Serum TOS concentrations did not change significantly over the course of the study in the control group. In contrast, the effect of measurement day was significant in both supplementation groups (*p* < 0.05). Between-group comparisons revealed a significant difference only on day 30, when TOS concentrations were lower in both supplementation groups than in the control group. Serum TAS concentrations remained unchanged throughout the study in the control and 0.5 mL/kg groups, whereas a significant effect of measurement day was observed in the 1 mL/kg group (*p* < 0.05). A significant between-group difference was observed only on day 30 (Table 3).

**Figure 1:**
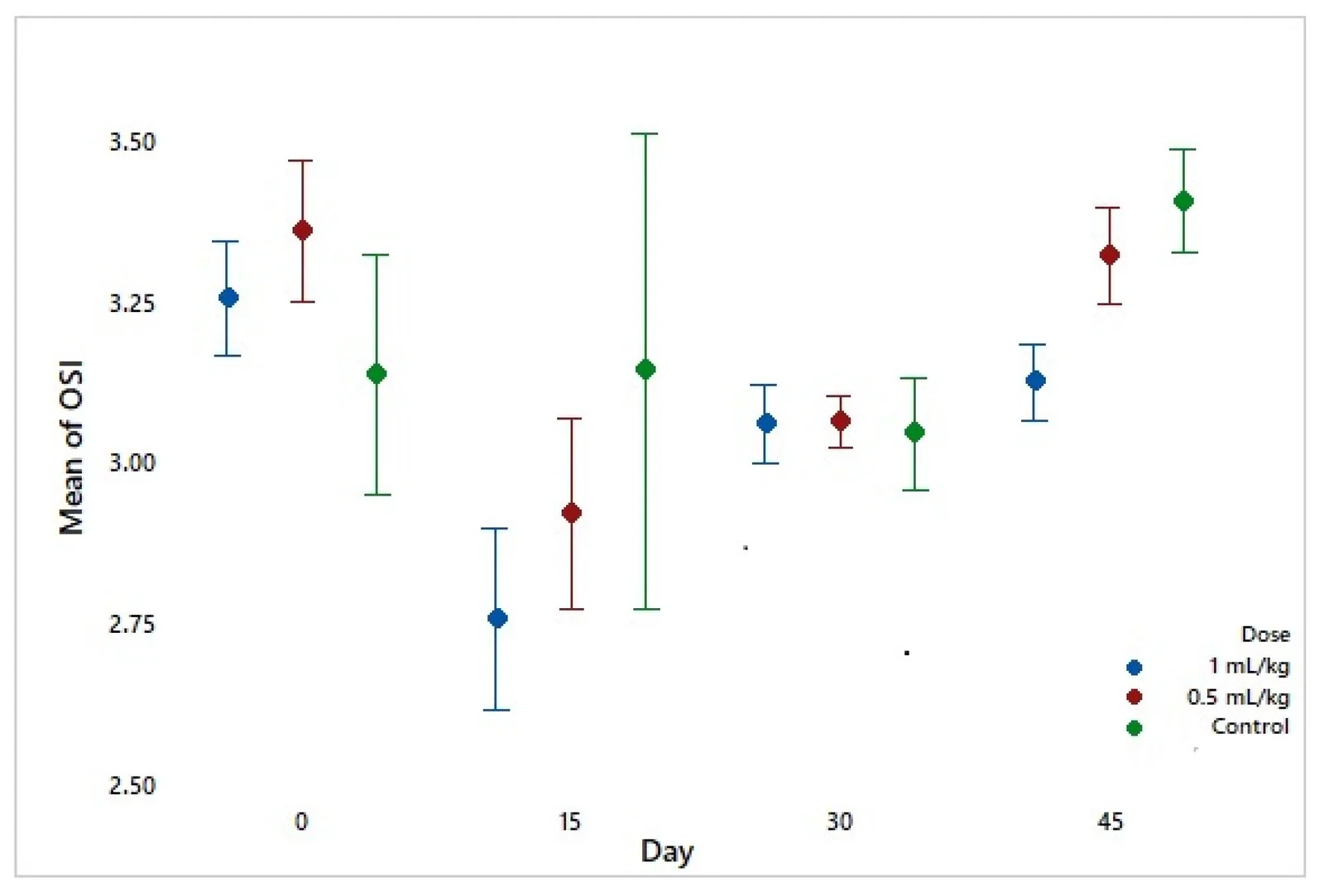
Temporal changes in serum oxidative stress index (OSI) values in the control (*n* = 9), 0.5 mL/kg PSO (*n* = 18), and 1 mL/kg PSO (*n* = 18) groups. Dots represent the mean values at each sampling time point, and error bars represent standard deviation (SD).

The effect of measurement day on OSI was not significant in the control group. In contrast, OSI values changed significantly over time in both supplementation groups (*p* < 0.05). Between-group comparisons revealed a significant difference only on day 45, when the OSI value was lower in the 1 mL/kg group than in the control group (Figure 1). The PSO dose × day interaction was not statistically significant for TOS, TAS, or OSI (*p* > 0.05; Table 3).

### 3.3. Growth Hormone and Immunoglobulin G

**Figure 2:**
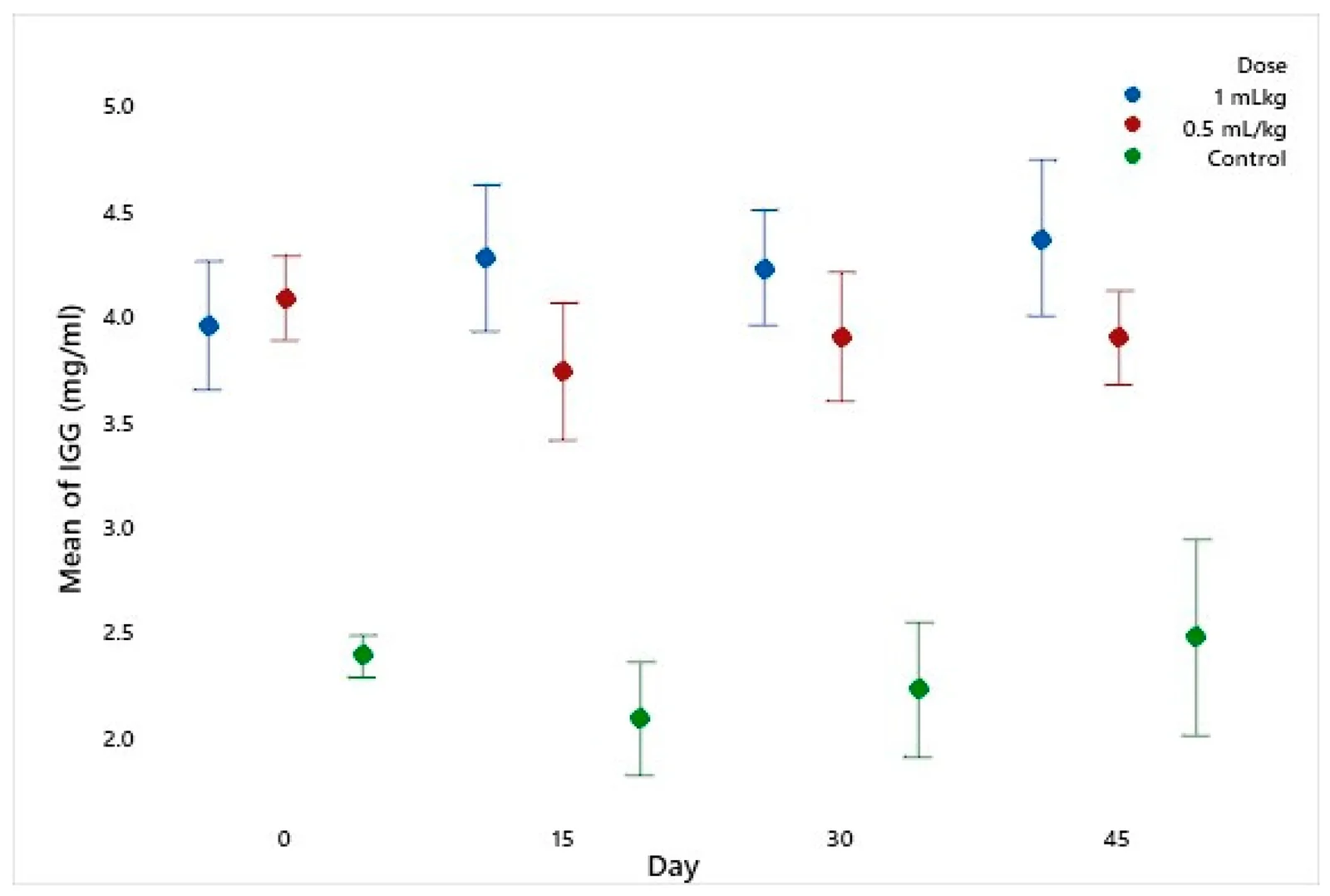
Temporal profiles of serum immunoglobulin G (IgG) concentrations in the control (*n* = 9), 0.5 mL/kg PSO (*n* = 18), and 1 mL/kg PSO (*n* = 18) groups. Dots represent the mean values at each sampling time point, and error bars represent standard deviation (SD).

Serum IgG concentrations were significantly higher in the supplementation groups than in the control group at all sampling time points (*p* ≤ 0.004; Figure 2). In contrast, neither the within-group effect of measurement day nor the PSO dose × day interaction was statistically significant. Serum GH concentrations did not differ significantly with respect to supplementation dose, measurement day, or the PSO dose × day interaction (Table 3).

### 3.4. Renal Function Indicators and Glucose

Serum BUN and urea concentrations changed significantly over time in all groups (*p* < 0.05). For both parameters, an increase was observed on day 15, followed by a decrease on day 45. Between-group comparisons revealed significant differences in BUN and urea concentrations on days 30 and 45. However, the PSO dose × day interaction was not statistically significant for either parameter (Table 4).

Serum creatinine concentrations changed significantly over time only in the 1 mL/kg group (*p* < 0.05). Between-group comparisons revealed a significant difference in creatinine concentrations on day 30. The PSO dose × day interaction was not significant for serum creatinine (Table 4).

Serum glucose concentrations changed significantly over time in all groups (*p* < 0.05). A significant between-group difference was detected only at baseline, whereas the PSO dose × day interaction was statistically significant (*p* < 0.05). At the end of the study, glucose concentrations were lower than their respective baseline values in all groups (Table 4).

### 3.5. Serum Liver Enzyme Activities and Total Protein Concentrations

Serum ALP activity changed significantly over time in the 0.5 and 1 mL/kg supplementation groups (*p* < 0.05). Between-group comparisons revealed a significant difference only on day 15. In addition, the PSO dose × day interaction was statistically significant for ALP (*p* < 0.05). Serum ALT and AST activities changed significantly over time only in the 1 mL/kg group (*p* < 0.05). However, neither between-group differences nor PSO dose × day interactions were statistically significant for either enzyme. Serum GGT activity did not change significantly among groups or over time during the study (Table 5).

A significant effect of measurement day on serum total protein concentration was observed in the 1 mL/kg group (*p* < 0.05). Although no significant between-group differences were detected, the PSO dose × day interaction was statistically significant (*p* < 0.05; Table 5).

### 3.6. Serum Lipid Profile

**Figure 3:**
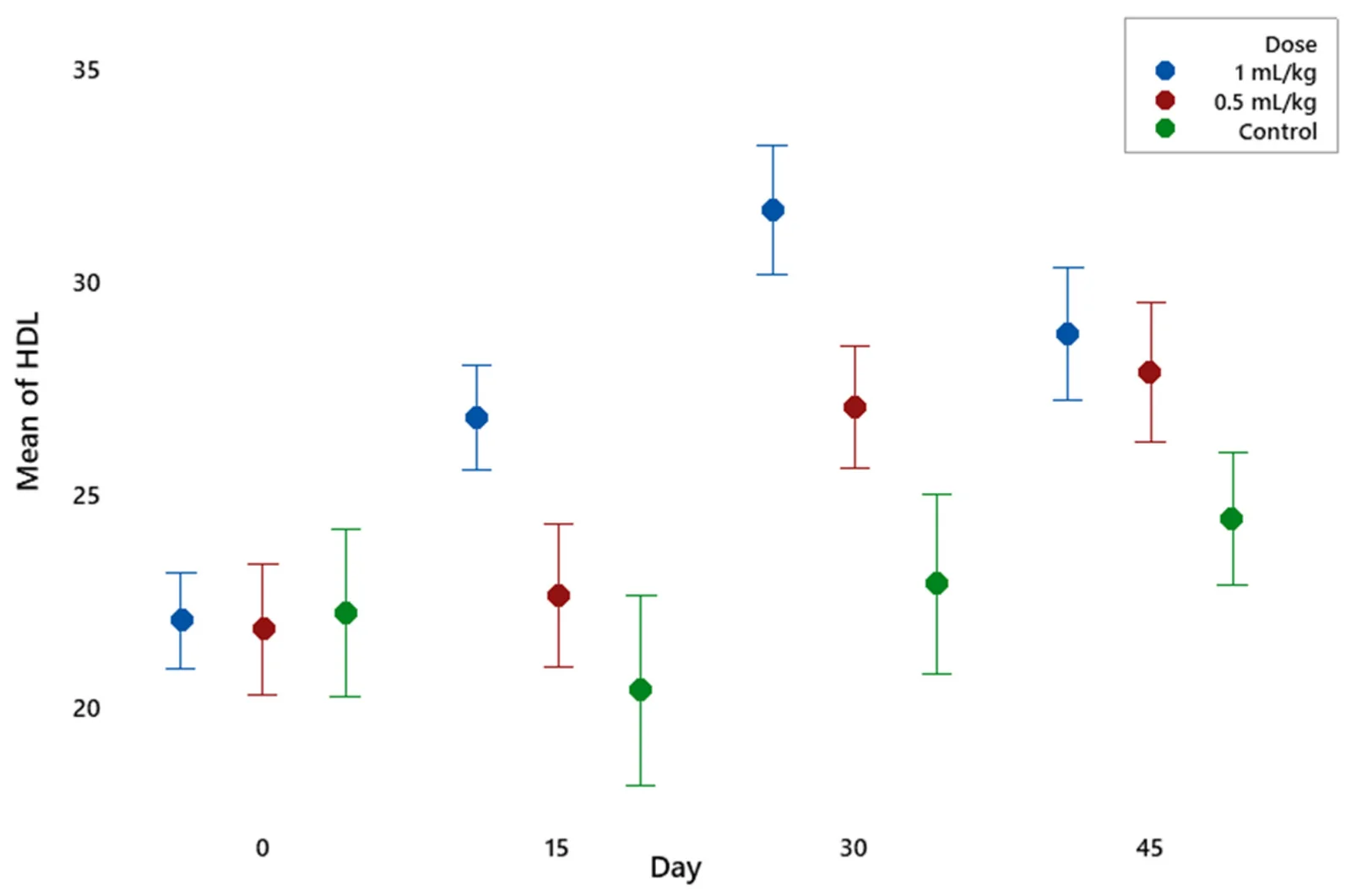
Temporal changes in serum high-density lipoprotein cholesterol (HDL) concentrations in the control (*n* = 9), 0.5 mL/kg PSO (*n* = 18), and 1 mL/kg PSO (*n* = 18) groups. Dots represent the mean values at each sampling time point, and error bars represent standard deviation (SD).

Serum HDL concentrations did not change significantly over the course of the study in the control group (*p* > 0.05). In contrast, the effect of measurement day was significant in both supplementation groups (*p* < 0.05). Between-group comparisons revealed significant differences in HDL concentrations on days 15 and 30, with higher concentrations in the 1 mL/kg group than in the control group. In addition, the PSO dose × day interaction was statistically significant for HDL (*p* < 0.05; Table 6; Figure 3). Serum LDL concentrations did not differ significantly among groups or change significantly over time during the study (Table 6).

Serum total cholesterol concentrations did not change significantly over time in the control group, whereas the effect of measurement day was significant in both supplementation groups (*p* < 0.05). However, no significant between-group differences were detected on any sampling day. The PSO dose × day interaction was statistically significant for total cholesterol (*p* < 0.05). Serum triglyceride concentrations changed significantly over time in all groups (*p* < 0.05). However, neither significant between-group differences nor a significant PSO dose × day interaction was observed (Table 6).

## 4. Discussion

In this study, the effects of oral administration of pumpkin seed oil (PSO) for 45 days on growth performance, immune response, oxidative stress, and selected serum biochemical parameters in lambs were evaluated. The findings indicate that PSO administration exerted beneficial effects, particularly on oxidative stress markers (TOS and OSI) and on serum IgG and HDL concentrations, whereas it did not significantly affect body weight gain or GH concentrations. These findings suggest that PSO may have potential as a functional dietary supplement for influencing selected oxidative and metabolic responses in lambs.

GC–FID analysis showed that the pumpkin seed oil used in the present study consisted primarily of linoleic acid (55.63%) and oleic acid (28.90%), with total PUFA, MUFA, and SFA proportions of 57.94%, 29.36%, and 11.45%, respectively (Table 1). This fatty acid profile is consistent with previous studies reporting that PSO is particularly rich in linoleic and oleic acids, which together constitute the majority of its fatty acid composition [10,11,37,38,39]. PUFAs have been reported to promote fatty acid β-oxidation and contribute to antioxidant defense mechanisms by activating signaling pathways that regulate lipid metabolism, particularly peroxisome proliferator-activated receptor-α (PPAR-α) [40,41]. However, because tocopherols, phenolic compounds, and other minor bioactive fractions were not directly analyzed in the present study, the observed biological effects cannot be attributed to a specific constituent. Nevertheless, a high PUFA content has been reported to contribute to the maintenance of cell membrane integrity, the limitation of oxidative damage, and the regulation of lipid metabolism [41,42]. Therefore, the changes observed in oxidative stress markers and the lipid profile may be associated with the characteristic fatty acid composition of PSO.

In the present study, the reductions observed particularly in TOS concentrations and OSI values in PSO-supplemented lambs support a potential oxidative stress–attenuating effect of PSO. TOS concentrations were lower in both supplementation groups than in the control group on day 30, and the OSI value was significantly lower in the 1 mL/kg group than in the control group on day 45 (Table 3; Figure 1). Importantly, the significant between-group differences were restricted to individual sampling time points and were not consistently maintained throughout the study. No consistent biological gradient was observed between the two supplementation doses. The transient nature of these responses may partly reflect physiological and homeostatic adaptation, particularly because the study was conducted in clinically healthy lambs with a relatively limited baseline oxidative burden. Because OSI simultaneously reflects the balance between oxidant and antioxidant systems, it is considered a more comprehensive indicator of oxidative stress than individual oxidant or antioxidant markers [22,24,26]. Studies reporting that PSO reduces lipid peroxidation and attenuates oxidative damage in different experimental animal models [19,27,42] are consistent with the lower TOS concentrations and OSI values observed in the present study. These effects may be associated with the characteristic fatty acid profile of PSO, which contains high proportions of linoleic acid, oleic acid, and other polyunsaturated fatty acids. In addition, tocopherols, particularly α- and γ-tocopherol, and phenolic compounds reported to be present in PSO may directly neutralize free radicals by interrupting the lipid peroxidation chain [13,14], thereby contributing to antioxidant defense [42]. However, because these bioactive constituents were not directly analyzed in the present study, the observed antioxidant effects cannot be attributed to any specific constituent. Therefore, the present findings suggest that PSO may be regarded not as a potent antioxidant agent that completely suppresses oxidative stress, but rather as a functional modulator that may contribute to the maintenance of redox homeostasis, with effects that may become more apparent over time (Table 3; Figure 1).

In the present study, serum IgG concentrations were higher in both PSO-supplemented groups (0.5 and 1 mL/kg) than in the control group at all sampling time points, suggesting that PSO may exert a supportive effect on processes associated with the humoral immune response (*p* ≤ 0.004; Table 3; Figure 2). Maintenance of adequate IgG concentrations during the post-neonatal period is considered important for resistance to infections [24] and overall immune competence in lambs [43]. Bioactive constituents present in PSO have been reported to influence antibody responses [44] and cellular protection [45,46]. In addition, n-3 PUFA, which is present in the fatty acid composition of PSO, has been suggested to possess direct immunomodulatory activity [47]. However, because IgG concentrations were already higher in the supplementation groups than in the control group at baseline, and neither the within-group effect of measurement day nor the dose × day interaction was significant, the observed differences cannot be interpreted as a strong response directly attributable to PSO supplementation. Furthermore, no significant differences in serum GH concentrations were detected with respect to supplementation dose, measurement day, or the dose × day interaction. This finding suggests that the observed immunological pattern may have occurred independently of GH-mediated mechanisms. The present findings indicate that serum IgG concentrations remained higher in the PSO-supplemented groups; however, given the between-group differences already present at baseline and the absence of significant effects of time or the dose × time interaction, this pattern cannot be attributed to a direct or robust immunostimulatory effect of PSO.

In the present study, PSO appeared to exert a selective modulatory effect on lipid metabolism, particularly through changes in HDL concentrations. While HDL concentrations remained stable in the control group throughout the study, they increased significantly over time in both PSO supplementation groups (0.5 and 1 mL/kg). In addition, HDL concentrations were higher in the 1 mL/kg group than in the control group on days 15 and 30. The significant day × dose interaction for HDL (pD × G = 0.006) suggests that the effect of PSO on HDL metabolism became more apparent over time (Table 6; Figure 3). In contrast, total cholesterol concentrations remained unchanged in the control group but showed an increasing trend over time in the supplementation groups. Although the day × dose interaction was significant (pD × G = 0.037), no significant between-group differences were detected at any sampling time point. Similarly, triglyceride concentrations increased over time in all groups, but no significant differences were observed among groups or in the day × dose interaction. LDL concentrations also did not differ significantly among groups throughout the study. The observed increase in HDL concentrations is generally consistent with previous studies reporting PSO’s antihyperlipidemic effects [48,49]. However, it should be taken into consideration that lipoprotein metabolism in growing ruminants differs from that in humans. In contrast, the time-related increases in total cholesterol and triglyceride concentrations observed in the present study do not fully correspond with the lipid-lowering effects reported in hyperlipidemic rat and mouse models [14,50]. However, in healthy, growing ruminants, physiological increases in total cholesterol and triglyceride concentrations may occur due to increased energy requirements and metabolic adaptation [43], and relatively high concentrations of these parameters have also been reported to reflect normal growth performance [51].

Accordingly, the changes in total cholesterol and triglyceride concentrations observed in the present study may reflect physiological metabolic adaptation associated with growth rather than an adverse effect specific to PSO supplementation. These changes in the lipid profile may be related to the high PUFA content of PSO, and linoleic acid has been reported to influence lipoprotein metabolism through PPAR-α-mediated pathways [41]. Overall, the present findings provide no evidence that PSO supplementation adversely affected lipid metabolism in lambs and indicate that it was associated with selective, time-dependent changes in lipoprotein metabolism, particularly given the observed increase in HDL concentrations (Table 6; Figure 3).

Serum glucose concentrations differed among groups at baseline but changed significantly over time in all groups (*p* < 0.05). On day 45, glucose concentrations were lower than their respective baseline values in all groups; however, the lowest value in the control group was recorded on day 15 (Table 4). A particularly pronounced decrease was observed in the 0.5 mL/kg group by the end of the study, which had the highest baseline glucose concentration. Previous studies have reported that pumpkin seed and PSO reduce serum glucose concentrations and insulin resistance in diabetic experimental models [14,15,52]. Similarly, a study in rabbits reported that PSO supplementation shifted glucose concentrations in stressed animals toward those observed in healthy controls [29]. In this respect, the findings of the present study are partially consistent with the previously reported regulatory effects of PSO on glucose metabolism. However, the presence of baseline differences in glucose concentrations among groups and the significant dose × day interaction (pD × G = 0.001) make it difficult to attribute the observed changes solely to the administered PSO dose. Therefore, although the present findings suggest that PSO supplementation may influence glucose homeostasis in healthy lambs, further studies assessing insulin sensitivity and glucose metabolism are required to clarify the underlying mechanisms.

When serum ALP, ALT, AST, and GGT activities and total protein concentrations were evaluated collectively, the findings suggested that PSO supplementation did not produce a consistent pattern indicative of adverse effects on hepatic function markers in lambs during the study period (Table 5). Previous studies have similarly reported that acute or subacute oral administration of pumpkin seed extract did not induce hepatic injury in healthy rat and pig models [53]. Likewise, oral administration of PSO to mice for 14 days was reported not to cause hepatic toxicity [28]. The findings of the present study are consistent with these reports. However, although no consistent adverse changes were observed in the serum hepatic biochemical indicators measured in the present study, no histopathological examination of liver tissue was performed; therefore, further studies are needed to establish PSO’s long-term hepatic safety.

Although body weight increased over time in all groups, no statistically significant differences were detected among groups. Similarly, serum GH concentrations did not differ significantly with respect to supplementation dose, measurement day, or the dose × day interaction. These findings indicate that, under the present experimental conditions and over the 45-day supplementation period, PSO had no evident effect on body weight gain or circulating GH concentrations in lambs. In contrast, when the changes observed in oxidative stress markers, serum IgG concentrations, and selected metabolic parameters are considered collectively, the biological effects of PSO may be related more closely to oxidative balance, immune-related processes, and metabolic regulation than to the growth axis.

The changes observed in BUN, urea, and creatinine concentrations during the study did not show a consistent pattern indicative of adverse effects on the measured serum renal biochemical indicators.

This study has several limitations. First, the presence of statistically significant between-group differences in IgG and glucose concentrations at baseline limits the extent to which the changes observed in these parameters during the study can be directly attributed to PSO supplementation. Second, only the fatty acid composition of the PSO used in the study was analyzed; tocopherols, phenolic compounds, and other minor bioactive constituents were not directly quantified. Therefore, the absence of direct quantification of these constituents limits the extent to which the observed biological effects can be attributed to specific bioactive constituents of PSO. Third, because the control group did not receive isocaloric or lipid-matched oil supplementation, the extent to which the observed metabolic and oxidative changes can be attributed specifically to PSO is limited, as these changes may also partly reflect additional dietary lipid, energy, and fatty acid intake from PSO. Fourth, the unequal group sizes, particularly the smaller number of animals in the control group, should be considered when interpreting the findings. Fifth, the 45-day experimental period does not allow conclusions regarding the long-term persistence of the observed effects. Future studies incorporating detailed phytochemical characterization of pumpkin seed oil, longer supplementation periods, an isocaloric or lipid-matched control group, and evaluation of potential mechanisms of action would contribute to a more comprehensive interpretation of the present findings.

## 5. Conclusions

Pumpkin seed oil supplementation did not significantly affect growth performance in lambs under the present experimental conditions. However, it was associated with selective changes in oxidative balance and lipid metabolism, without a consistent pattern indicative of adverse hepatic or renal effects. Serum IgG concentrations remained higher in the supplementation groups throughout the study; however, the presence of baseline differences limits direct attribution of this finding to PSO supplementation. Overall, these findings suggest that pumpkin seed oil may have potential as a functional dietary supplement for influencing redox homeostasis and selected metabolic processes in lambs. To the best of our knowledge, this study is among the first experimental investigations to comprehensively evaluate the effects of pumpkin seed oil on growth performance, oxidative status, serum IgG concentrations, and biochemical parameters in small ruminants. However, further studies are needed to elucidate the underlying mechanisms of action in greater detail.

## Figures and Tables

**Table 1:** Fatty acid composition of pumpkin seed oil.

Fraction	Fatty Acid	Abbreviation	Area (%)
SFA	Myristic acid	C14:0	0.10612
SFA	Pentadecanoic acid	C15:0	0.01833
SFA	Palmitic acid	C16:0	7.73435
SFA	Heptadecanoic acid	C17:0	0.04497
SFA	Stearic acid	C18:0	3.30205
SFA	Tricosanoic acid	C23:0	0.08214
SFA	Lignoceric acid	C24:0	0.15976
MUFA	Palmitoleic acid	C16:1	0.10067
MUFA	cis-10-Heptadecenoic acid	C17:1	0.03551
MUFA	Elaidic acid	C18:1 n-9t	0.01579
MUFA	Oleic acid	C18:1 n-9c	28.90164
MUFA	cis-11-Eicosenoic acid	C20:1 n-9	0.21488
MUFA	Nervonic acid	C24:1 n-9	0.09332
PUFA	Linoleic acid	C18:2 n-6c	55.63027
PUFA	γ-Linolenic acid	C18:3 n-6	0.34790
PUFA	α-Linolenic acid	C18:3 n-3	1.10519
PUFA	cis-11,14-Eicosadienoic acid	C20:2	0.01820
PUFA	cis-8,11,14-Eicosatrienoic acid	C20:3 n-6	0.34269
PUFA	Unidentified PUFA	-	0.49584
Calculated totals	ΣSFA	-	11.44772
	ΣMUFA	-	29.36181
	ΣPUFA	-	57.94009
	Unidentified peaks	-	1.25038
	Total area (%)	-	100.00

SFA, saturated fatty acids; MUFA, monounsaturated fatty acids; PUFA, polyunsaturated fatty acids. Individual fatty acids and calculated totals are expressed as relative peak area (%).

**Table 2:** Body weights of the lambs during the study.

Day	Group	*n*	Body Weight (kg)	Minimum (kg)	Maximum (kg)
0	Control	9	37.80 ± 12.11	21.40	56.60
	0.5 mL/kg	18	38.76 ± 9.45	23.00	57.60
	1 mL/kg	18	36.21 ± 9.24	21.00	58.40
15	Control	9	41.44 ± 13.20	23.60	62.40
	0.5 mL/kg	18	42.72 ± 9.76	26.80	62.40
	1 mL/kg	18	40.51 ± 9.13	25.20	60.40
30	Control	9	45.29 ± 13.43	25.80	65.20
	0.5 mL/kg	18	47.67 ± 9.38	32.80	65.40
	1 mL/kg	18	45.58 ± 9.42	29.60	66.60
45	Control	9	48.24 ± 12.49	30.00	66.00
	0.5 mL/kg	18	50.83 ± 9.02	36.40	67.20
	1 mL/kg	18	48.84 ± 9.22	33.20	69.20

Values are presented as mean ± SD.

**Table 3:** Serum oxidant–antioxidant and ELISA values.

Parameter	Group	Day 0	Day 15	Day 30	Day 45	*p*_Day_
GH (pg/mL)	Control	1902.00 ± 363.00	1836 ± 470	1870.00 ± 356.00	1767.00 ± 289.20	0.887
	0.5 mL/kg	1732.20 ± 378.00	1789.50 ± 339.40	1984.00 ± 890.00	1863.00 ± 595.00	0.612
	1 mL/kg	1594.00 ± 452.00	1803.60 ± 366.70	1590.50 ± 394.80	1603.00 ± 591.00	0.435
	*p*_dose_	0.186	0.955	0.182	0.367	*p*_D×G_ = 0.268
IgG (mg/mL)	Control	2.38 ± 0.30 ^b^	2.09 ± 0.81 ^b^	2.22 ± 0.96 ^b^	2.47 ± 1.39 ^b^	0.833
	0.5 mL/kg	4.08 ± 0.85 ^a^	3.73 ± 1.38 ^a^	3.90 ± 1.30 ^a^	3.89 ± 0.95 ^a^	0.840
	1 mL/kg	3.95 ± 1.29 ^a^	4.27 ± 1.47 ^a^	4.22 ± 1.16 ^a^	4.36 ± 1.57 ^a^	0.827
	*p*_dose_	0.000 *	0.001 *	0.001 *	0.004 *	p_D×G_ = 0.855
TOS (µmol H_2_O_2_ equiv./L)	Control	57.98 ± 16.35	60.40 ± 23.02	61.37 ± 5.98 ^a^	62.91 ± 6.26	0.912
	0.5 mL/kg	60.56 ± 11.05 ^A^	52.60 ± 9.63 ^B^	57.18 ± 2.37 ^b,AB^	62.84 ± 7.83 ^A^	0.003 *
	1 mL/kg	55.64 ± 7.46 ^AB^	50.82 ± 10.15 ^B^	56.43 ± 2.79 ^b,AB^	59.21 ± 3.90 ^A^	0.004 *
	*p*_dose_	0.420	0.216	0.004 *	0.165	*p*_D×G_ =0.402
TAS (mmol Trolox equiv./L)	Control	1.89 ± 0.66	1.97 ± 0.70	2.03 ± 0.29 ^a^	1.85 ± 0.21	0.885
	0.5 mL/kg	1.84 ± 0.51	1.82 ± 0.26	1.87 ± 0.12 ^ab^	1.89 ± 0.20	0.916
	1 mL/kg	1.72 ± 0.23 ^B^	1.86 ± 0.20 ^AB^	1.85 ± 0.13 ^b,AB^	1.90 ± 0.17 ^A^	0.029 *
	*p*_dose_	0.585	0.634	0.031 *	0.803	*p*_D×G_ = 0.572
OSI (AU)	Control	3.15 ± 0.56	3.15 ± 1.11	3.05 ± 0.26	3.41 ± 0.24 ^a^	0.670
	0.5 mL/kg	3.37 ± 0.47 ^A^	2.93 ± 0.63 ^B^	3.07 ± 0.17 ^AB^	3.33 ± 0.31 ^ab,A^	0.008 *
	1 mL/kg	3.26 ± 0.38 ^A^	2.76 ± 0.60 ^B^	3.07 ± 0.26 ^AB^	3.13 ± 0.25 ^b,A^	0.003 *
	*p*_dose_	0.485	0.439	0.979	0.029 *	*p*_D×G_ = 0.515

Data are presented as mean ± SD. Different lowercase letters indicate differences among groups within the same day, and different uppercase letters indicate differences among days within the same group (*p* < 0.05). *p*_Day_: day effect; *p*_dose_: dose effect; *p*_D×G_: day × dose interaction. * *p* < 0.05.

**Table 4:** Serum blood urea nitrogen (BUN), urea, creatinine, and glucose concentrations.

Parameter	Group	Day 0	Day 15	Day 30	Day 45	*p*_Day_
BUN (mg/dL)	Control	18.86 ± 1.97 ^BC^	23.72 ± 3.66 ^A^	20.73 ± 1.88 ^ab,AB^	15.99 ± 2.7 ^ab,C^	0.001 *
	0.5 mL/kg	17.34 ± 3.25 ^B^	23.38 ± 4.08 ^A^	22.51 ± 3.49 ^a,A^	16.45 ± 3.67 ^a,B^	0.001 *
	1 mL/kg	16.44 ± 2.48 ^B^	21.71 ± 3.11 ^A^	19.63 ± 2.87 ^b,A^	13.99 ± 2.08 ^b, C^	0.001 *
	*p*_dose_	0.109	0.275	0.021 *	0.043 *	*p*_D×G_ = 0.332
UREA (mg/dL)	Control	40.32 ± 4.16 ^BC^	50.78 ± 7.85 ^A^	44.33 ± 40 ^ab,AB^	34.19 ± 5.78 ^ab,C^	0.001 *
	0.5 mL/kg	37.11 ± 6.96 ^B^	50.02 ± 8.72 ^A^	48.20 ± 7.47 ^a,A^	35.17 ± 7.85 ^a,B^	0.001 *
	1 mL/kg	35.17 ± 5.32 ^B^	46.42 ± 6.66 ^A^	42.02 ± 6.12 ^b,A^	29.96 ± 4.44 ^b,C^	0.001 *
	*p*_dose_	0.110	0.269	0.021 *	0.044 *	*p*_D×G_ = 0.330
Creatinine (mg/dL)	Control	0.49 ± 0.09	0.48 ± 0.1	0.48 ± 0.10 ^b^	0.57 ± 0.11	0.207
	0.5 mL/kg	0.47 ± 0.12	0.46 ± 0.09	0.51 ± 0.08 ^b^	0.54 ± 0.09	0.056
	1 mL/kg	0.53 ± 0.11 ^B^	0.53 ± 0.08 ^B^	0.60 ± 0.08 ^a,A^	0.60 ± 0.09 ^A^	0.021 *
	*p*_dose_	0.227	0.077	0.003 *	0.174	*p*_D×G_ = 0.297
Glucose (mg/dL)	Control	73.56 ± 6.98 ^b,AB^	68.33 ± 6.93 ^B^	79.87 ± 4.83 ^A^	70.00 ± 6.38 ^B^	0.003 *
	0.5 mL/kg	83.78 ± 7.66 ^a,A^	73.61 ± 7.42 ^B^	77.87 ± 6.95 ^AB^	65.44 ± 7.56 ^C^	0.001 *
	1 mL/kg	78.83 ± 6.25 ^ab,A^	71.82 ± 7.76 ^B^	82.50 ± 6.50 ^A^	66.41 ± 8.66 ^B^	0.001 *
	*p*_dose_	0.003 *	0.236	0.108	0.361	*p*_D×G_ = 0.001 *

Data are presented as mean ± SD. Different lowercase letters indicate differences among groups within the same day, and different uppercase letters indicate differences among days within the same group (*p* < 0.05). *p*_Day_: day effect; *p*_dose_: dose effect; *p*_D×G_: day × dose interaction. * *p* < 0.05.

**Table 5:** Serum liver enzyme and total protein values.

Parameter	Group	Day 0	Day 15	Day 30	Day 45	*p*_Day_
ALP (U/L)	Control	298.2 ± 95.3	229.2 ± 68.2 ^b^	331.10 ± 75.60	255.1 ± 81.8	0.055
	0.5 mL/kg	387.1 ± 123.5 ^A^	245.9 ± 63.1 ^b,B^	311.70 ± 102.60 ^AB^	246.7 ± 95 ^B^	0.001 *
	1 mL/kg	379.7 ± 128.7 ^A^	307 ± 86.1 ^a,AB^	343.80 ± 91.70 ^AB^	267.5 ± 77.7 ^B^	0.007 *
	*p*_dose_	0.177	0.017 *	0.589	0.767	*p*_D×G_ = 0.010 *
ALT (U/L)	Control	35.7 ± 47.1	37.9 ± 38.1	36.47 ± 22.52	47.7 ± 41.7	0.902
	0.5 mL/kg	34.43 ± 27.14	59.4 ± 71.1	37.21 ± 19.47	64.9 ± 79.6	0.261
	1 mL/kg	21.27 ± 10.27 ^B^	39.1 ± 47.6 ^AB^	41.49 ± 22.9 ^AB^	74.6 ± 60.9 ^A^	0.002 *
	*p*_dose_	0.280	0.496	0.786	0.614	*p*_D×G_ = 0.357
AST(U/L)	Control	105.8 ± 44.5	150.4 ± 106.2	138.1 ± 49.9	215.2 ± 194.1	0.255
	0.5 mL/kg	147.6 ± 76.9	234.4 ± 225.5	169.6 ± 72	244.9 ± 224.5	0.231
	1 mL/kg	106.34 ± 36.51 ^B^	179 ± 144.2 ^AB^	181.6 ± 69.8 ^AB^	300.8 ± 247.4 ^A^	0.002 *
	*p*_dose_	0.072	0.452	0.295	0.612	*p*_D×G_ = 0.550
GGT (U/L)	Control	62.66 ± 26.3	66.1 ± 16.6	64.73 ± 7.29	68.90± 12.63	0.889
	0.5 mL/kg	74.33 ± 13.09	74.06 ± 15.77	76.77 ± 17.29	73.24 ± 14.35	0.910
	1 mL/kg	64.12 ± 12.87	69.96 ± 15.83	70.94 ± 15.48	70.68 ± 14.5	0.463
	*p*_dose_	0.110	0.460	0.150	0.730	*p*_D×G_ = 0.351
Total protein (g/dL)	Control	6.34 ± 0.83	5.94 ± 0.47	6.03 ± 0.37	6.3 ± 0.39	0.319
	0.5 mL/kg	6.43 ± 0.48	6.17 ± 0.39	6.22 ± 0.49	6.4 ± 0.48	0.227
	1 mL/kg	6.01 ± 0.47 ^B^	6.02 ± 0.39 ^B^	6.35 ± 0.35 ^A^	6.20 ± 0.33 ^AB^	0.035 *
	*p*_dose_	0.076	0.339	0.178	0.344	*p*_D×G_ = 0.008 *

Data are presented as mean ± SD. Different lowercase letters indicate differences among groups within the same day, and different uppercase letters indicate differences among days within the same group (*p* < 0.05). *p*_Day_: day effect; *p*_dose_: dose effect; *p*_D×G_: day × dose interaction. * *p* < 0.05.

**Table 6:** Serum lipid concentrations.

Parameter	Group	Day 0	Day 15	Day 30	Day 45	*p*_Day_
Total cholesterol (mg/dL)	Control	41.6 ± 17.94	38.48 ± 9.53	40.21 ± 9.43	46.26 ± 6.56	0.539
	0.5 mL/kg	38.09 ± 13.17 ^B^	39.39 ± 13.81 ^AB^	44.92 ± 12.41 ^AB^	50.52 ± 13.45 ^A^	0.024 *
	1 mL/kg	35.81 ± 8.9 ^B^	43.98 ± 8.38 ^AB^	47.38 ± 11.07 ^A^	47.67 ± 11.25 ^A^	0.002 *
	*p*_dose_	0.543	0.351	0.312	0.613	*p*_D×G_ = 0.037 *
Triglycerides (mg/dL)	Control	28.18 ± 18.54 ^AB^	17.85 ± 7.54 ^B^	16.79 ± 2.94 ^B^	31.49 ± 12.06 ^A^	0.026 *
	0.5 mL/kg	23.64 ± 9.82 ^B^	18.73 ± 9.38 ^B^	18.57 ± 7.14 ^B^	32.75 ± 12.53 ^A^	0.001 *
	1 mL/kg	21.23 ± 7.05 ^B^	23.1 ± 10.35 ^AB^	19.98 ± 7.38 ^B^	29.08 ± 6.95 ^A^	0.006 *
	*p*_dose_	0.322	0.275	0.501	0.577	*p*_D×G_ = 0.129
LDL (mg/dL)	Control	15.11 ± 4.57	15.56 ± 3.83	15.31 ± 3.11	19.33 ± 3.86	0.084
	0.5 mL/kg	16.04 ± 6.96	14.9 ± 6.85	17.21 ± 6.46	19.78 ± 6.46	0.164
	1 mL/kg	14.26 ± 4.54	15.31 ± 4.8	16.08 ± 3.42	16.3 ± 4.56	0.504
	*p*_dose_	0.642	0.952	0.603	0.129	*p*_D×G_ = 0.246
HDL (mg/dL)	Control	22.2 ± 5.88	20.39 ± 6.65 ^b^	22.88 ± 6.3 ^b^	24.41 ± 4.69	0.552
	0.5 mL/kg	21.81 ± 6.54 ^B^	22.61 ± 7.12 ^ab,AB^	27.04 ± 6.1 ^ab,AB^	27.86 ± 6.92 ^A^	0.014 *
	1 mL/kg	22.03 ± 4.81 ^B^	26.8 ± 5.16 ^a,AB^	31.67 ± 6.27 ^a,A^	28.77 ± 6.57 ^A^	0.001 *
	*p*_dose_	0.985	0.034 *	0.004 *	0.251	*p*_D×G_ = 0.006 *

Data are presented as mean ± SD. Different lowercase letters indicate differences among groups within the same day, and different uppercase letters indicate differences among days within the same group (*p* < 0.05). *p*_Day_: day effect; *p*_dose_: dose effect; *p*_D×G_: day × dose interaction. * *p* < 0.05.

## Data Availability

The original contributions presented in this study are included in the article. Further inquiries can be directed to the corresponding author.
